# Surgery

**Published:** 1894-03

**Authors:** 


### SURGERY.

EDITED BY DR. F. W. McRAE.

### ANTISEPSIS IN RAILWAY SURGERY.

On this subject Dr. C. B. Stemen has an interesting article in the Fort Wayne *Journal of Medical Sciences* for November. He says:

It is to be presumed that all surgeons of the present day are familiar with the principles and methods of aseptic and antiseptic practice, and yet we believe it proper, in fact important, to present the various steps necessary in complying with their observance. In the practice of railway surgery we find our patients covered with dust, perhaps on the roadside, or in some old car, maybe a passenger coach, or baggage, or freight car, either of which is far from being aseptic or a proper place in which to make a surgical operation, either minor or capital. But the patient may present such a condition as to demand prompt action. What should the surgeon do under such circumstances? He should, if possible, give the patient the benefit of prompt action. If the patient can be transported to his home or hospital, he should be prepared for removal. If a large blood vessel has been lacerated and there is danger of hemorrhage, there should be immediate action to arrest the hemorrhage, and the wound covered over with some antiseptic gauze, or better sprinkle with iodoform, aristol or subnit. of bismuth. A towel should be wrung out of a bichloride solution 1-1000 or a five per cent. of carbolic acid, and then with a few turns of a roller held in place; this will prevent septic germs from entering the wounds while being transported.

The question may be asked: Where will the surgeon obtain the towel and the solution of carbolic acid, or bichloride of mercury? We answer that every railway surgeon should have all of these things in his emergency case, which he is expected always to have with him. There is no excuse for any surgeon to fail for want of any of these precautions.

After the patient has been received at his home or hospital, and the injury is to be dressed permanently, whether it be a lacerated wound, or whether it be a thigh or shoulder amputation, it matters not, the precautions are the same. The operator and his assistants should thoroughly cleanse their hands and forearms with soap and water, and the nail brush. After the use of the soap and water, the hands should be rubbed with sulphuric ether, and then placed into a solution of the bichloride of mercury. Neither the operator nor his assistants should handle anything after their hands have been prepared. An abundance of hot water should always be at hand, and the making of the antiseptic solution be intrusted to a competent nurse or assistant, and a number of basins filled with these solutions should be at hand for the purpose of immersing the hands, and for irrigating the wound. I should have said that the parts to be operated on should be first shaved, and then cleansed the same as the hands of the surgeon, with the flesh brush, soap and water, ether and bichloride solution, and as soon as cleansed, they should be covered over with a towel wrung out of a solution of bichloride.

In many cases it becomes very important to have the parts very thoroughly scrubbed with soap and water, and then use the flesh brush. I have observed this scrubbing and cleansing of parts to be operated on in the hospitals in Vienna and other European hospitals, where it was so thorough that the cuticle was nearly removed. In the clinics of the great surgeon Billroth in Vienna, the most thorough cleansing of the parts is practiced, and the use of antiseptics is most scrupuously carried out. Neither the operator nor dresser is expected to handle anything that has not been sterilized. The instruments should be boiled for thirty minutes, provided there is the time, and then placed in a five per cent. solution of carbolic acid, and each instrument as it is required should be removed from the bath by the surgeon himself, or handed him by an assistant whose hands have been prepared, and carried directly to the wound, and when it is no longer needed should again be immersed. Under no circumstances should an instrument be laid on the operating table, or any other table or bed, and then used again. This rule must be enforced and insisted upon by every surgeon who hopes to be successful, or succeed in antiseptic practice, and I may say that this one rule is frequently violated, especially in a tedious and prolonged operation. During the manipulations of the wound it should at short intervals be copiously irrigated with the sublimate or carbolic solution. Whenever it is practicable this irrigation should be continuous. I prefer the solution of carbolic acid, to that of the bichloride, for the reason that it does not tarnish the instruments, and where there is a large surface to be irrigated, there is some danger of sufficient mercury being absorbed to cause some constitutional disturbance, and result in severe *tenesmus* and *abdominal* pains. A word of caution in regard to the use of a strong solution of carbolic acid. The strength should not be more than two and one-half per cent. when used for irrigating a wound. A fountain syringe, which has been thoroughly cleansed, is a good vehicle for conveying the irrigating solutions, and in order that the solution can be ready, several large pitchers full should be close at hand to refill the fountain as needed.

### SPONGES.

The best sponges are made by taking absorbent cotton, and antiseptic gauze placed in a basin with hot carbolized or sublimated water and covered over with a clean towel. If sea sponges are used great care should be taken that they are thoroughly cleansed and placed in a five per cent. solution of carbolic acid. Sponges having been once used on suppurating wounds should not be used again.

### BASINS.

These should be porcelain or earthen, so that the solution will not effect them, and should be filled with hot water, either carbolized or sublimated, and be near at hand, so that the operator could immerse his hands when necessary. I shall not take up your time by giving the methods for preparing ligatures, only to say that I prefer the sterilized silk, and believe it to be the safest ligature, and in most cases I do not pay any attention to it after tying the artery. I cut it short and let it alone, and up to this time have not had any bad results following this procedure. I have used catgut for a long time, but have had a few cases of secondary hemorrhage, which I know resulted from the too early absorption of the catgut. I prefer the sterilized silk and believe it to be the most reliable.

SUTURES.

We may employ catgut, silkworm gut, horse hair or sterilized silk, but do not employ adhesive plasters for the purpose of approximating the edges of a wound. The sutures should be placed with the needles intended to be used in a five per cent. solution of carbolic acid. Before approximating the edges of the wound, and thus closing it, all capillary oozing should stop and the wound be as nearly dry as possible. The stitches should not be too tight, for several reasons, one is that it greatly increases the suffering of the patient, and still another, that it is a very frequent cause of inflammation.

I will not enter into the discussion of drainage-tubes, only to say this, that when an operation has been delayed for some time after the injury, and there is considerable of swelling and accumulation of serum in the soft parts, a drainage-tube made from decalcified bone, or a rubber tube which has been sterilized, may, with some advantage, be used, but, as a rule, I do not use them, and believe that they are quite frequently employed when they do more harm than good. After the sutures have been placed and the parts evenly approximated, then the wound should again be irrigated and all blood stains carefully removed, and the wound sprinkled over with aristol, or what I have been using mostly for the past year is one part of aristol and three parts of subnit. of bismuth. This I have thoroughly mixed, and use by sprinkling it over the wound and then applying the antiseptic gauze, and of this I prefer the bichloride gauze, and next the carbolized, but I have no use for the iodoform gauze, for the reason that in so many cases it has caused a very troublesome and annoying vesicular eruption. Let the parts be well covered with gauze, and then some absorbent cotton and bandages, and place the parts to rest, and allow the dressing to remain ten days or a fortnight, unless some symptoms should arise indicating that there was some inflammation or suppuration going on in the wound, when the dressing should at once be removed, but under the same antiseptic precautions as when the first dressing was applied, irrigating the parts with the antiseptic solution and the same precautions in preparing the hands of the surgeon and assistants.

### ABSCESS.

Never try fluctuation *across* a limb, always *along* it.

Never forget that:

1. Abscesses near a large joint often communicate with the joint.
2. Abscesses near a large artery sometimes communicate with the artery.
3. Abdominal wall abscesses sometimes communicate with the gut or the solid viscera.

Never forget that *early* openings are imperative in abscesses situated:

1. In the neighborhood of joints.
2. In the abdominal wall.
3. In the neck, under the deep fascia.
4. In the palm of the hand.
5. Beneath periosteum.
6. About the rectum, prostate, and urethra.

To wait for abscesses to "point" or to "burst" in these situations is culpable as well as cowardly.

Remember the frequency with which hematomata and traumatic aneurism have been mistaken for abscesses, and incised with untoward results.

Do not open an abscess anywhere near a large artery without first using a stethoscope, and then only by Hilton's method (*i. e.*, scalpel, director and dressing forceps).

Never, under any circumstances, use for *exploratory* puncture "that surgical abomination—a grooved needle"—for it will allow contamination of all the tissues through which it brings the fluids (Thornton).

Never plunge in opening abscesses; never squeeze the sac after doing so.

Do not forget that your incision should radiate:

1. In abscesses pointing near the nipple.
2. In abscesses near the anus.
3. In scarifying the chemosis of the cornea.

And that your incision should be longitudinal:

1. In the hand.
2. In the urethra.
3. On the vertex.

Do not forget that incisions for abscesses in neck and face should run parallel with the wrinkles and folds.

Do not be afraid of hurting the lacteal tubes in mammary abscess. More harm is done to the gland by the enlargement of the walls of the abscess than by a free incision.

Never make a palmar incision except in the middle of the lower third and in the axial line of the fingers or at the sides of the palm.

Do not forget, in opening a deep abscess in the lumbar region without the projection of the abscess, to cut down opposite a transverse process, not between them, for fear of wounding a lumbar artery.—*Fenwick.*

---

### THE RETENTION OF URINE AFTER OPERATIONS.

Guepin (*Gaz. de Hopitaux,* No. 33, 1893) says that retention of urine after operation may occur at any age from puberty on. It is especially noted after abdominal operations or those on the lower extremities.

There are two clinical forms, an indolent and a painful form. The first appears when the organs are quite healthy, and the latter in those who have diseased urinary organs. The duration of the retention varies from eight, ten or twenty-four hours to three days. The cause of this condition is supposed to be due to a swelling of the mucous membrane of the urethra. According to the author, however, there are three causes:

(1) Spasm of the urethra, especially of the pars prostatica.

(2) Paralysis of the bladder.

(3) The abnormal position of the patient, as upon the back.

In the treatment of this condition the patient should be told to stand up when possible; if this fail, warm clysma might be given. When this fails the catheter should be used.—*University Medical Magazine.*

### THE TREATMENT OF HEMORRHOIDS.

Reclus ( *Gaz. de Hopitaux*, No. 35, 1893 ) speaks of the use of hot water for the relief of painful hemorrhoidal tumors. Tampons, wet with a two per cent. cocaine solution, should be kept in contact with the anus. In order to give a more certain relief the anus should be dilated with a bivalve speculum, with or without anesthesia. A tampon wet with a two per cent. cocaine solution is inserted in the ampulla recti for two or three minutes. An injection of a one per cent. solution of cocaine is made directly into the substance of the sphincter muscle. The speculum is then introduced and maximum dilatation made.

Reclus has treated sixty cases after this method with only one failure. The relief was absolute, and the condition did not return. The author has never seen any disagreeable or dangerous symptoms result from this treatment. If it is thought likely that there will be a return of the trouble, the pile tumors should be cut off with cocaine anesthesia.—*University Medical Magazine.*

